# Correlates of Breakthrough SARS-CoV-2 Infections in People with HIV: Results from the CIHR CTN 328 Study

**DOI:** 10.3390/vaccines12050447

**Published:** 2024-04-23

**Authors:** Cecilia T. Costiniuk, Terry Lee, Joel Singer, Yannick Galipeau, Corey Arnold, Marc-André Langlois, Judy Needham, Mohammad-Ali Jenabian, Ann N. Burchell, Hasina Samji, Catharine Chambers, Sharon Walmsley, Mario Ostrowski, Colin Kovacs, Darrell H. S. Tan, Marianne Harris, Mark Hull, Zabrina L. Brumme, Hope R. Lapointe, Mark A. Brockman, Shari Margolese, Enrico Mandarino, Suzanne Samarani, Bertrand Lebouché, Jonathan B. Angel, Jean-Pierre Routy, Curtis L. Cooper, Aslam H. Anis

**Affiliations:** 1 Division of Infectious Diseases and Chronic Viral Illness Service, McGill University Health Centre, Royal Victoria Hospital—Glen Site, Montreal, QC H4A 3J1, Canada; suzanne.samarani@muhc.mcgill.ca (S.S.); bertrand.lebouche@mcgill.ca (B.L.); jean-pierre.routy@mcgill.ca (J.-P.R.); 2 Infectious Diseases and Immunity in Global Health Program, Research Institute of the McGill University Health Centre, Montreal, QC H4A 3J1, Canada; 3 Department of Experimental Medicine, McGill University, Montreal, QC H4A 3J1, Canada; 4 CIHR Canadian HIV Trials Network (CTN), Vancouver, BC V6Z 1Y6, Canada; tlee@hivnet.ubc.ca (T.L.); jneedham@advancinghealth.ubc.ca (J.N.); shari.margolese@gmail.com (S.M.); emandarino@rogers.com (E.M.); aslam.anis@ubc.ca (A.H.A.); 5 Centre for Advancing Health Outcomes, St. Paul’s Hospital, Vancouver, BC V6Z 1Y6, Canada; 6 School of Population and Public Health, University of British Columbia, Vancouver, BC V6T 1Z3, Canada; 7 Department of Biochemistry, Microbiology and Immunology, University of Ottawa, Ottawa, ON K1N 6N5, Canada; ygali093@uottawa.ca (Y.G.); carnold3@uottawa.ca (C.A.); langlois@uottawa.ca (M.-A.L.); jangel@ohri.ca (J.B.A.); 8 Department of Biological Sciences, Université du Québec à Montréal, Montreal, QC H2X 1Y4, Canada; jenabian.mohammad-ali@uqam.ca; 9 Department of Family and Community Medicine, St. Michael’s Hospital, Unity Health Toronto, Toronto, ON M5B 1W8, Canada; ann.burchell@unityhealth.to; 10 Dalla Lana School of Public Health, University of Toronto, Toronto, ON M5T 3M7, Canada; catharine.chambers@mail.utoronto.ca; 11 Faculty of Health Sciences, Simon Fraser University, Burnaby, BC V5A 1S6, Canada; hasina.samji@bccdc.ca (H.S.); zbrumme@sfu.ca (Z.L.B.); mark_brockman@sfu.ca (M.A.B.); 12 British Columbia Centre for Disease Control, Vancouver, BC V5Z 4R4, Canada; 13 MAP Centre for Urban Health Solutions, St. Michael’s Hospital, Unity Health Toronto, Toronto, ON M5B 1T8, Canada; darrell.tan@unityhealth.to; 14 Division of Infectious Diseases, Department of Medicine, University of Toronto, Toronto, ON M5S 3H2, Canada; sharon.walmsley@uhn.ca; 15 Clinical Sciences Division, Department of Immunology, Li Ka Shing Knowledge Institute, St. Michael’s Hospital, University of Toronto, Toronto, ON M5B 1T8, Canada; mario.ostrowski@gmail.com; 16 Division of Infectious Diseases, Faculty of Medicine, University of Toronto, Toronto, ON M5S 3H2, Canada; ckovacs@mlmedical.com; 17 Institute of Public Health Policy, Management and Evaluation, Dalla Lana School of Public Health, University of Toronto, Toronto, ON M5S 3M6, Canada; 18 British Columbia Centre for Excellence in HIV/AIDS, Vancouver, BC V6Z 1Y6, Canada; mharris@bccfe.ca (M.H.); mhull@bccfe.ca (M.H.);; 19 Department of Molecular Biology and Biochemistry, Faculty of Science, Simon Fraser University, Burnaby, BC V5A 1S6, Canada; 20 Department of Family Medicine, Faculty of Medicine and Health Sciences, McGill University, Montreal, QC H3S 1Z1, Canada; 21 Division of Infectious Diseases, Department of Medicine, Ottawa Hospital Research Institute, University of Ottawa, Ottawa, ON K1H 8L6, Canada; ccooper@toh.ca; 22 Division of Hematology, Department of Medicine, McGill University Health Centre, Montreal, QC H4A 3J1, Canada

**Keywords:** COVID-19 vaccination, SARS-CoV-2, HIV, breakthrough infection, immunogenicity, humoral immunity, immunosuppressed hosts

## Abstract

COVID-19 breakthrough infection (BTI) can occur despite vaccination. Using a multi-centre, prospective, observational Canadian cohort of people with HIV (PWH) receiving ≥2 COVID-19 vaccines, we compared the SARS-CoV-2 spike (S) and receptor-binding domain (RBD)-specific IgG levels 3 and 6 months post second dose, as well as 1 month post third dose, in PWH with and without BTI. BTI was defined as positivity based on self-report measures (data up to last study visit) or IgG data (up to 1 month post dose 3). The self-report measures were based on their symptoms and either a positive PCR or rapid antigen test. The analysis was restricted to persons without previous COVID-19 infection. Persons without BTI remained COVID-19-naïve until ≥3 months following the third dose. Of 289 participants, 92 developed BTI (31.5 infections per 100 person-years). The median days between last vaccination and BTI was 128 (IQR 67, 176), with the most cases occurring between the third and fourth dose (n = 59), corresponding to the Omicron wave. In analyses adjusted for age, sex, race, multimorbidity, hypertension, chronic kidney disease, diabetes and obesity, a lower IgG S/RBD (log10 BAU/mL) at 1 month post dose 3 was significantly associated with BTI, suggesting that a lower IgG level at this time point may predict BTI in this cohort of PWH.

## 1. Introduction

HIV infection induces both humoral and cell-mediated immune defects, impacting the severity of respiratory infections, as well as vaccine-induced immune responses [1]. During the COVID-19 pandemic, people with HIV (PWH) had twice the risk of hospitalization compared to HIV-negative individuals [2], and large studies showed up to a threefold increased risk of COVID-19-related mortality in PWH [3,4,5,6,7]. The vulnerability of PWH to severe COVID-19 outcomes stems from a combination of risk factors including high rates of smoking, multimorbidity [8] and social determinants of health [9,10]. Importantly, the suboptimal immunogenicity to many common vaccines in PWH is well recognized and may be attributed, in part, to ongoing B cell dysfunction and ongoing immune activation, resulting in inappropriate responses to stimuli [11,12,13]. HIV infection, especially in the context of low CD4 T cell counts (<200 cells/mm^3^) and unsuppressed viral loads [14,15], may cause predisposition to poor immunogenicity to various vaccines such as influenza [16], pneumococcal [17,18], meningococcal [19] and hepatitis A [20,21,22] and B vaccines [23,24]. Additional vulnerabilities increase the risk of SARS-CoV-2 acquisition and symptomatic/severe COVID-19 in PWH, including multimorbidity and membership in socioeconomic or racialized groups disproportionately affected by COVID-19 [25]. As the majority of PWH enrolled in COVID-19 vaccine trials have had normal CD4 T cell counts (>500 cells/mm^3^) and few comorbidities [26,27], these immunogenicity results may not be generalizable to the wide spectrum of PWH who are followed in Canadian centres. 

Despite advances in understanding the immunogenicity outcomes in PWH, there remains a paucity of data regarding COVID-19 breakthrough infection (BTI) in PWH. The objective of the current study was to compare the socioeconomic, clinical and vaccine immune response correlates of BTI following two and three doses of COVID-19 vaccines in a prospective Canadian cohort of PWH enrolled in a vaccine immunogenicity study (CTN 328). This knowledge could help to identify persons at risk for BTI and inform us on whether different vaccine strategies, such as additional vaccine boosters for certain individuals, may portend benefit.

## 2. Materials and Methods

The CTN 328 study was designed and implemented in 2020 to address the knowledge gaps regarding the COVID-19 vaccine responses in diverse PWH and HIV-negative participants over long time periods, when little information was available on the responses to third or booster vaccinations in PWH. It was also designed to describe the safety and tolerability of COVID-19 vaccines in PWH [28]. An exploratory objective was to determine whether subpopulations of PWH respond differently to COVID-19 vaccination [28]. The CTN 328 was a multi-centre, prospective, observational cohort study of PWH recruited from sites in Montreal, QC, Toronto, ON, Ottawa ON, and Vancouver, BC with the aim of comparing their COVID-19 vaccine-induced immune response with that of HIV-negative controls. The CTN 328 protocol was previously published [28] and later amended to accommodate third and fourth doses/boosters. Enrolment occurred from April to June 2021 in Vancouver and from June 2021 to January 2022 for the remaining sites [29,30]. The participants attended visits prior to and following the vaccine dosing for phlebotomy and questionnaire completion [29,30].

### 2.1. Ethics Statement

This study was conducted in accordance with the Declaration of Helsinki and approved by the McGill University Health Centre Research Ethics Board (REB) under protocol 2022-7857, the Ottawa Health Science Network REB under protocol 20210361-01H, the University Health Network REB under protocol 21-5504 and the University of British Columbia Providence Health Care and Simon Fraser University REBs under protocols #H21-01515 and #H21-00742. Written informed consent was obtained from all the participants. 

### 2.2. Human Participants

The inclusion criteria for participation in the CTN 328 study were (must meet all criteria) (1) age ≥16 years; (2) living with HIV (for the PWH group); (3) planning on receiving a COVID-19 vaccine or has received no more than 2 doses at enrolment; (4) able to provide signed, informed consent; (5) able to attend study visits. The exclusion criteria included signs or symptoms of active COVID-19 at enrolment [28]. 

Subpopulations of PWH were prioritized for the study enrolment: (1) Older age (≥55 years) given its association with immunosenescence, comorbidities and reduced vaccine efficacy [31,32]; (2) Immune non-responders (CD4 T cell count < 350 cells/mm^3^, CD4/CD8 < 0.75 with an undetectable viral load for at least 6 months). Immune non-responders may be at risk of more adverse COVID-19-related outcomes than HIV immune responders [7,33]; (3) Multimorbidity (defined as having ≥2 comorbidities) given the association with worse COVID-19 outcomes with COVID-19 [34]; (4) An HIV-positive ‘stable’ or ‘reference’ group (undetectable HIV viral load for at least 6 months, CD4 T cell counts ≥ 350 cells/mm^3^ and a maximum of one comorbidity). The sites were encouraged to enrol PWH at risk of worse outcomes due to COVID-19 infection, such as the subpopulations described above [28,29], because at-risk participants had been excluded from many of the vaccine trials that had been reported. The current analysis was restricted to persons who received 2 or more vaccine doses 

### 2.3. Study Visits

For the present analysis, we evaluated samples collected between 11 August 2021 and 20 April 2022 at 3 and 6 months after the 2nd dose and 1 month after the 3rd dose. Moderately to severely immunocompromised individuals in some provinces were given a 3rd dose [35] as part of the primary series. Of note, in the 3 provinces enrolling participants, PWH were not included in this group. In accordance with the Public Health Agency of Canada guidelines, the 3rd doses of the mRNA-1273 vaccine were 50 µg (vs. 100 µg 1st and 2nd doses) for those under 65 years [36,37], whereas the 3rd doses of the BNT162b2 vaccine were the same as for the 1st and 2nd doses (30 µg).

### 2.4. Sociodemographic Health and Clinical History

Sociodemographic and general health information were captured via participant self-reporting. The data were gathered from interviews and clinic chart reviews following written informed consent and included comorbidities, medications, body mass index and history and date of COVID-19 infection. COVID-19 infection was based on a positive PCR test and/or self-report with a rapid antigen test (RAT). With regards to HIV history, we extracted year of HIV diagnosis, nadir and most recent CD4 T cell counts and ART regimen [28,29]. We also collected contextual information related to risk factors, including working in an environment in close proximity to others, working in a hospital or healthcare facility, the number of individuals living in the participant’s household and the number of bedrooms and bathrooms in the household.

### 2.5. Definition of BTI and Clinical Evaluation for COVID-19 Infection

BTI was defined as positivity based on the self-reporting (data up to the last study visit) or IgG data (only available up to 1 month post dose 3). Thus, all the BTI after 1 month post dose 3 was based on self/site reporting. The self-reporting was based on symptoms and either a positive PCR or rapid antigen test. All participants who developed symptoms consistent with COVID-19 throughout the study were asked to go to their nearest testing centre for testing. Participants were asked to notify the study staff by telephone of the test result so that this could be recorded. 

COVID-19-like symptoms were defined as a temperature >38.0 °C associated with any one or more of the following clinical symptoms: feverishness/chills; cough; tachypnea/dyspnea; wheezing/stridor; rhinorrhea; sore throat; myalgias; loss of smell or taste; diarrhea. Participants were asked to record the signs and symptoms of COVID-19-like illness daily until resolution. These signs and symptoms are outlined in Appendix A. Hospitalizations, as disclosed by the participant or clinic records, were recorded. 

### 2.6. Sample Collection

At each visit, blood was collected to isolate serum and plasma [28,29]. 

### 2.7. Humoral Immunity (SARS-CoV-2 Binding Antibodies)

The levels of IgG targeting the SARS-CoV-2 spike trimer (S) protein, spike receptor-binding domain (RBD) and nucleocapsid (N) protein were measured using an automated high-throughput chemiluminescent ELISA [38,39]. Vaccine-induced immunity was distinguished by co-positivity with the S and RBD protein and infection-induced immunity by co-positivity with the S and N protein (signal-to-cutoff ratio ≥ 1.0) [38] This assay was selected since it has been used in other Canadian studies [40,41,42], enabling future comparison of the results across cohorts.

If someone was anti-N-seropositive but an infection was not self/site-reported, they were still included in the BTI group. However, the timing of infection was uncertain in this case, so the sample date for anti-N seropositivity was used as the infection date.

For those with anti-N seropositivity after dose 2 (without self-reported infection), they were only counted as having a BTI if they had a negative anti-N result after dose 2 prior to anti-N seropositivity. This way, we could be sure that the infection occurred after dose 2. We excluded cases that did not satisfy this criterion. The IgG antibody titres (binding antibody units (BAU)/mL) were generated using a conversion model (4-parameter log-logistic curve based on measurements from the WHO International Standard) [43].

### 2.8. Statistical Analysis

All the analyses were performed using Statistical Analysis System (SAS) software version 9.4 (SAS Institute, Cary, NC, USA). We examined the differences in the proportions of participants with BTI vs. without BTI between a stratified sub-population of interest (persons ≥ 55 years old, immune non-responders (CD4 < 350 cells/mm^3^, CD4/CD8 ratio < 0.75, UD VL) and the HIV reference population (CD4 ≥ 350 cells/mm^3^, UD VL and ≤1 comorbidity). We also examined the differences in the proportions of PWH developing BTI based on having received the ChAdOx1 vaccine as at least one of the first two vaccine doses and those who received mRNA vaccines for both doses. These comparisons were based on the Chi-square test or Fisher’s exact test as appropriate. Other continuous variables, such as age and CD4 count, were compared between groups using the Wilcoxon rank sum test. Descriptive statistics were used to determine number of days between the last vaccine dose and BTI. We also examined the difference in the IgG spike/RBD (log10 BAU/mL) levels at 3 and 6 months post dose 2 (±2 months) and 1 month post dose 3 (±2 weeks) in participants with vs. without BTI after these time points using the Wilcoxon rank sum test. Only IgG data prior to breakthrough infection were included. We further conducted logistic regression adjusted for age, sex, multimorbidity, hypertension, chronic kidney disease, diabetes and obesity to examine the effect of IgG spike/RBD on the odds of BTI. We did not adjust for vaccine type since it was believed that vaccine type would be too highly collinear with IgG to be included as an adjustment variable. No adjustment for multiple comparisons was made given the exploratory nature of the study. *p* values < 0.05 were considered statistically significant.

### 2.9. Data Availability

The data presented in this study may be made available upon written request to the corresponding author. 

## 3. Results

### 3.1. Participant Characteristics Stratified by BTI

A total of 375 PWH were enrolled in the CTN 328 study, of whom 289 (77.1%) individuals were included in the current analysis. Thus, all BTI 1 month post dose 3 was based on self/site reporting. The self-reporting was based on symptoms and either a positive PCR or rapid antigen test. Of 289 participants, 92 (31.8%) developed BTI, equating to 31.5 infections per 100 person-years. Individuals were excluded if they had COVID-19-like symptoms and PCR-confirmed COVID-19 infection prior to their second vaccine dose (n = 44/375, 11.7%); if the timing of COVID-19 infection was unconfirmed (n = 25/375, 6.7%); if these infections were identified only using IgG data and it could not be confirmed that the infection occurred after dose 2; if they did not receive dose 2 or if they were lost to follow-up following dose 2 (n = 17/375, 4.5%). The median number (IQR) of days between last vaccination and BTI was 128 (67, 176), with most of the cases occurring after the third dose and before the fourth dose (n = 59). 

The baseline characteristics for PWH with (n = 92) and without (n = 197) BTI are presented in Table 1 and Supplementary Table S1. 

An undetectable HIV viral load 6 months preceding enrolment was observed in PWH without vs. with BTI (91% vs. 87%, *p* = 0.279), respectively. Of the individuals with BTI, 4 individuals (2%) were not on ART, whereas all the individuals without BTI were on ART. In both groups, greater than two-thirds of the PWH were on integrase strand inhibitor regimens (73.1 vs. 73.9%), and the ART regimen did not significantly differ between groups (*p* = 0.052).

The most frequent comorbidities included obesity (20.4% vs. 20.7% without vs. with BTI, respectively, *p* = 0.961), dyslipidemia (15.5% vs. 12% without vs. with BTI, respectively, *p* = 0.420) and hypertension (16.1% vs. 8.7% without vs. with BTI, respectively, *p* = 0.091), but these did not differ significantly between those without and those with BTI (Supplementary Table S1). The participants with vs. without BTI did not differ in terms of their age, sex, ethnicity, duration of HIV, CD4 or nadir CD4 count or comorbidities. There were no significant differences in the proportions of participants with BTI vs. without BTI who were persons ≥ 55 years old, immune non-responders (CD4 < 350 cells/mm^3^, CD4/CD8 ratio < 0.75, UD VL) or in the HIV reference population (CD4 ≥ 350 cells/mm^3^, UD VL and ≤1 comorbidity). 

### 3.2. Numbers and Types of Vaccines Received Stratified by BTI

The vast majority of the participants had received mRNA vaccines. In the current analysis, performed in September 2023, 83% of PWH without BTI and 89% with BTI had received at least two mRNA vaccines (either BNT162b2 and/or mRNA-1273). The types of vaccines received and the time interval between vaccine doses are shown in Table 2. More individuals with BTI had received ChAdOx1 vaccines, as one or both of the first two doses, than individuals who had received mRNA vaccines for both doses (*p* = 0.004). 

### 3.3. SARS-CoV-2 Antibody Concentrations Stratified by BTI

**Figure 1:**
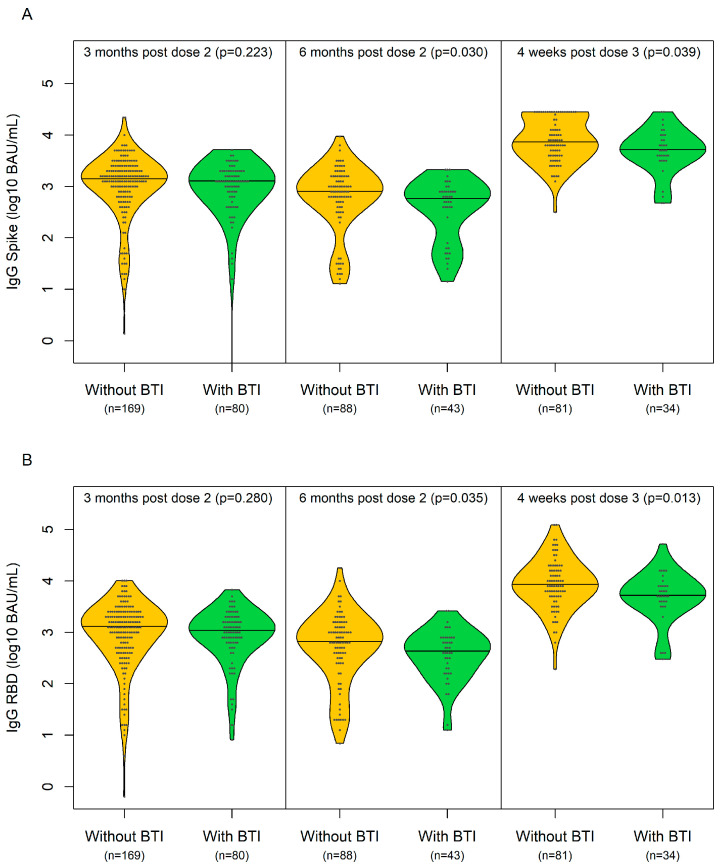
Median (IQR) IgG spike/RBD (log10 BAU/mL) at 3 months post dose 2 (±1 month), 6 months post dose 2 (±2 months) and 1 month post dose 3 (±2 weeks) in participants without (yellow) and with (green) breakthrough infection. (**A**) Median (IQR) IgG spike (log10 BAU/mL); (**B**) median (IQR) IgG RBD (log10 BAU/mL).

The median titres of IgG spike/RBD (log10 BAU/mL) at 3 months post dose 2 (±1 month), months post dose 2 (±2 months) and 1 month post dose 3 (±2 weeks) are depicted in Figure 1 and Table 3. Only serology data prior to breakthrough infection were included. In the analyses adjusted for age, sex, race, multimorbidity, hypertension, chronic kidney disease, diabetes and obesity, a lower IgG spike/RBD value (log10 BAU/mL) at 1 month post dose 3 was significantly associated with BTI in PWH. 

### 3.4. Clinical Presentation of Participants with BTI

Most of the participants with BTI had mild symptoms (Table S2), encompassing fever, cough, sore throat, malaise, headache, muscle pain, nausea, vomiting, diarrhea and/or loss of taste/smell [44]. Shortness of breath was seen in 12 individuals (17.4%) with BTI for whom their symptoms were captured, suggesting that a nearly a fifth of individuals had moderate infection [44]. We did not capture data on abnormal chest imaging, which is also suggestive of moderate infection. Three participants were hospitalized (Table S3). We did not capture information that enabled us to differentiate between severe illness (SpO_2_ < 94% on room air, ratio of arterial partial pressure of oxygen to fraction of inspired oxygen (PaO_2_/FiO_2_) < 300 mm Hg, a respiratory rate > 30 breaths/min or lung infiltrates > 50%) and critical illness (respiratory failure, septic shock and/or multiple organ dysfunction) [44]. None of the participants died.

## 4. Discussion

In this study, we examined BTI arising in a large cohort of PWH in Canada enrolled in a prospective observational study on COVID-19 vaccine immunogenicity. BTI occurred at a rate of 31.5 infections per 100 person-years in the PWH. Whereas other studies examining BTI in PWH have been conducted over the course of the pandemic, most of these studies have examined BTI based on HIV status (i.e., HIV-positive vs. HIV-negative). These studies yielded variable results. Furthermore, some studies have found that the severity of COVID-19 illness was similar between PWH and HIV-negative individuals [45,46,47,48], whereas other studies have found increased severity amongst PWH compared to HIV-negative persons [49,50,51,52,53]. One study which examined the risk of COVID-19 hospitalization in the pre-Omicron era found that PWH had about a two times greater risk of COVID-19 hospitalization than HIV-negative individuals in crude analyses, which was attenuated in propensity-score-weighted models [2]. This study found the risk differential could be explained by sociodemographic factors and history of comorbidity, underscoring the need to address the social and comorbid vulnerabilities (e.g., injecting drugs) that are more prominent among PWH [2]. The discrepancies across studies have been attributed to small sample sizes, a lack of HIV-negative control groups, the confounding inherent in observational studies or them having been conducted prior to the COVID-19 vaccine rollout. Moreover, finding an ideal control group remains a challenge for HIV studies since this group would, in theory, be individuals identical to the HIV-seropositive group in all aspects except for HIV status [54]. 

A study conducted by Coburn et al. combined electronic-health-record-based cohort data from integrated health systems and academic centres to examine PWH fully vaccinated prior to 30 June 2021 and HIV-negative individuals matched by date of full vaccination, age, race and ethnicity and sex. Among 113,994 patients (33,029 PWH and 80,965 HIV-negative controls) followed until 31 December 2021 [55], the risk of severe illness (defined as hospitalization within 28 days following SARS-CoV-2 BTI, with a primary or secondary COVID-19 discharge diagnosis) was low for both groups (6.8% of 3649 vaccinated PWH and HIV-negative individuals) and did not differ by HIV status overall [55]. BTI was 28% higher in the PWH than the HIV-negative persons (adjusted hazard ratio, 1.28 [95% CI, 1.19–1.37]) [55]. Most of the people with BTI were aged 55 or older (60%) and male (89%) [55]. In another study by Sun et al., cohort study data from the National COVID Cohort Collaborative (N3C), using a centralized electronic-medical-record-based repository of COVID-19 clinical data from academic medical centres across the US, were analyzed [56]. Amongst persons with HIV who had received a full vaccination series between December 2020 and September 2021, the IR of breakthrough infection was 7.1 (95% CI, 7.1–7.2) per 1000 person-months for people without immune dysfunction vs. 9.1 (95% CI, 8.8–9.4) per 1000 person-months for person with HIV infection [56]. Furthermore, HIV infection was independently associated with an increased BTI rate compared to persons without HIV or another form of immune compromise [56]. Importantly, the associations were independent of demographic characteristics, geographic region and comorbidity burden.

We found that the PWH who developed BTI were similar to the PWH who did not develop BTI with regards to age, sex, ethnicity, HIV duration, CD4 count and comorbidities. Although our study did not examine the severity of BTI, Coburn et al. found that severe breakthrough infection was 59% higher in the PWH with CD4 counts less than 350 cells/mm^3^ compared to the HIV-negative controls (aHR, 1.59; 95% CI, 0.99 to 2.46; *p* = 0.049) [55]. Furthermore, in multivariable analyses among PWH, Coburn et al. found that being female and older was associated with an increased risk of BTI. Other possible reasons for the differences between our study and the previously published study may relate to the types of individuals included in these studies and economic disparities. As regards vaccine type, we observed that the PWH who received ChAdOx1 vaccines as either one or two of their first two doses had significantly more BTIs than those who received mRNA vaccines for both doses. This finding has been observed in other studies [57]. mRNA vaccines are recognized for their ability to elicit strong immune responses compared to non-mRNA vaccines [58].

A very small number of participants in our study who developed BTI were hospitalized, and nobody died. These findings suggest that vaccination appeared to attenuate the disease severity in those acquiring infection. In the study by Coburn et al., mechanical ventilation and death were rare among PWH with BTI. When mechanical ventilation and death did occur, it was more likely in persons over 55 years of age with one or more comorbidity [55]. Although our study was not designed to assess the severity of COVID-19 infection amongst persons with BTI, these findings are nonetheless reassuring. 

Interestingly, we found that PWH with BTI had a significantly lower median IgG spike/RBD level at 1 month post dose 3 than the participants without BTI. This observation suggests that lower IgG may predict BTI at this time point in this cohort. Although studies in both PWH and HIV-negative populations have tried to identify the protective antibody level against BTI, few have identified a specific cutoff [59]. 

In a systematic review of COVID-19 vaccine antibody responses in PWH which included studies from 1 January 2020 to 31 March 2022, Chun et al. reviewed 28 studies from 12 countries. Twenty-two (73%) studies reported high COVID-19 vaccine seroconversion rates in PWH, although PWH with lower baseline CD4 counts and CD4/CD8 ratios and higher baseline viral loads had lower seroconversion rates and antibody titres [60,61]. To date, there is still no consensus on what constitutes the best correlate of immune protection, and there are no disease-specific correlates of immune protection. In a large prospective longitudinal population-based study of COVID-19 in the general population, Vivaldi et al. observed correlations between vaccine-induced anti-spike antibody titres and protection against severe BTI [57]. Several studies have demonstrated that higher levels of neutralizing antibodies are associated with immune protection from symptomatic BTI infection following vaccination [62,63,64,65,66].

As IgG is involved in binding viruses, it makes intuitive sense there may be an association between lower IgG levels preceding BTI, although neutralizing antibody titres during the first months after vaccination may be better correlated with vaccine effectiveness [62,66] and have been predictive of the risk of BTI [62,65,67]. However, no specific antibody or neutralizing threshold titre has yet been identified that can predict the degree of protection, as it changes over time with waning or boosting. Moreover, it is worth underscoring that rates of BTI are a consequence of an individual’s level of immunity at a particular moment in time, the variant to which a person is exposed and the severity of the illnesses [67] BTI is influenced by, including their timing, frequency, severity and degree of infectiousness. BTI is important, as it is informative regarding the duration of restrictions in order to limit transmission and the need for additional booster doses or changes to dosing intervals [67].

SARS-CoV-2 vaccines have been designed to elicit immune responses—including the formation of antibodies against the outer spike protein of the Ancestral strain. However, SARS-CoV-2 variants, including Omicron, harbour mutations in the spike protein. Omicron harbours greater than 30 mutations in its virus’ spike protein, which attaches to cells, thereby increasing the chance of infection [68]. Moreover, Omicron and other variants are more transmissible than the Ancestral strain and are more able to evade antibody response, resulting in more BTIs [69]. Antibody waning, along with antigenic drift, limits the ability of long-term immunity to prevent reinfection. Furthermore, while earlier variants had a few mutation amino acids in their RBDs, Omicron harbours 15 RBD mutations—many in key antibody sites—preventing antibody binding. In addition, while earlier variants required both ACE2 and TMPRSS2 proteins to inject their genomes into a cell, Omicron bound only to ACE2. As many cells do not have TMPRSS2 on their exterior, if the virus does not need the surface protein, it has a wider number of potential target cells. Furthermore, unlike Delta, which had a subdued interferon response, Omicron activated interferon signaling [70]. Omicron infections tended to be less severe, but they were also associated with rises in cases and significant increases in hospitalizations and deaths. Therefore, Omicron and its subvariants quickly became dominant starting in late 2021, as they were more transmissible and also evaded antibodies more than prior variants. Consequently, there was an increase in BTIs [70,71,72].

Although this is not included in the current manuscript, more research needs to be dedicated to understanding the role of cell-mediated immunity and mucosal immunity in the nasal, oropharyngeal and pulmonary tissues in relation to protection against SARS-CoV-2 and other emerging viral infections [69,73,74,75,76]. In vaccinated HIV-negative individuals infected with SARS-CoV-2 during the Omicron wave, there were increased spike-specific responses during the infection of vaccinated compared to unvaccinated individuals [73]. Spike-specific clusters of CD4 T cells and plasmablasts expanded, and CD8 T cells were potently activated during the first week. In contrast, memory B cell activation, neutralizing antibody production and the primary responses to non-spike antigens occurred during the second week. 

Given the importance of different arms of the immune system working synergistically, it is likely that the best correlate of protection will be a combination of measures. HIV induces a wide spectrum of immune abnormalities and is characterized by chronic low-grade inflammation despite ART Cell-mediated immunity [61,74,75,76,77,78,79] and antibody-dependent cell cytotoxicity merit examination, especially given the underlying propensity of HIV to infect and target CD4 T cells, which play a role in establishing vaccine memory, and to induce dysfunction in cytotoxic CD8 T cell response [80]. 

Measures of mucosal immunity should also be given greater attention given that this is the first site of immune defense. In HIV-negative individuals, it has been demonstrated that a local secretory component-associated IgA response is induced by COVID-19 mRNA vaccination that persists in some, but not all, individuals. The serum and saliva IgA responses were modestly correlated at 2–4 weeks post dose 2. Interestingly, the levels of anti-spike serum IgA (but not IgG) at this time point were lower in the participants who subsequently become infected with SARS-CoV-2 [81].

Our study has several limitations. An important limitation is the risk of misclassification bias. During many time periods during the pandemic, PCR tests were reserved for healthcare workers, hospitalized persons and residents in long-term care facilities. During other time periods, rapid antigen tests were in short supply in some regions. Therefore, participants may have had symptoms, but diagnostic testing may not have been easily accessible. Ascertainment of BTI was based on patient-initiated healthcare seeking, as opposed to being systematic. Thus, there may have been cases of asymptomatic BTI that were not captured. We acknowledge that relying on self-report and IgG data may introduce bias, as BTI in those vaccinated or who have had a prior infection may result in mild symptoms or even a lack of symptoms. Self-reporting can also impact representativeness and accuracy due to self-selection and recall biases. Moreover, there was a considerable overlap in the antibody levels between both those with and without BTI such that one cannot identify a threshold level of antibody that predicts protection. We did not determine the duration of BTI, nor the duration of post COVID chronic symptomatology. Furthermore, we did not report on the neutralization efficiency, as neutralization assays are still in progress at this time. The relative lack of information on disease severity is another limitation. This study was conducted in Canada, which has a well-developed healthcare system, and therefore its findings may not be generalizable to PWH in other countries. Even within Canada, the recruitment sites were large urban tertiary care clinics, and the participants may not necessarily be representative of other PWH in rural regions who differ in their access to healthcare, adherence to preventative behaviours and social determinants of health. Furthermore, our findings may not be generalizable to all the SARS-CoV-2 variants in circulation given that our study was conducted when the Alpha, Delta and Omicron variants were circulating. Given the timing of most of the BTIs in our study (between the third and fourth vaccine doses), most of the BTIs occurred when Omicron was circulating.

## 5. Conclusions

In summary, BTIs were more common in participants who received ChAdOx1 vaccines as the primary series. BTI was not associated with age or HIV-related factors. PWH with BTI following COVID-19 vaccination had lower median IgG Spike/RBD antibody levels 1 month post dose 3 than those who did not develop BTI at this point, suggesting that antibody levels at this time point may be a predictor of BTI in this cohort. However, given the large overlap in the IgG spike/RBD antibody levels between groups, this statement requires some caution in its interpretation. Ongoing evaluation is needed to determine the optimal correlates of immune protection and the durability of immune response in order to further inform vaccine guidelines and policies.

## Figures and Tables

**Table 1:** Participant characteristics stratified based on COVID-19 BTI.

Variable	Participants without COVID-19 BTI (n = 197)	Participants with COVID-19 BTI (n = 92)	*p*
Age			0.098
Median (IQR)	54.8 (45.1, 62.8)	53.6 (39.7, 61.7)	
Range	(19.7, 81.6)	(24.3, 83.5)	
# Missing	1 (0.5%)	0 (0%)	
Sex, n (%)			0.786
Male	150 (76.1)	73 (79.3)	
Female	45 (22.8)	19 (20.7)	
Prefer to self-describe	2 (1.0)	0 (0.0)	
Self-declared race or ethnicity ^1^, n (%)			0.125
White	118 (60.2)	60 (65.9)	
Black	38 (19.4)	9 (9.9)	
Other	40 (20.4)	22 (24.2)	
Unknown	1	1	
Sub-population, n (%)			
Age ≥ 55 years	97/196 (49.5)	39/92 (42.4)	0.261
Multimorbidity (≥2 comorbidities)	56/191 (29.3)	23/92 (25.0)	0.448
Immune non-responder ^2^	18/187 (9.6)	7/85 (8.2)	0.713
HIV + stable/reference (CD4 ≥ 350, suppressed VL and ≤1 comorbidity)	96/183 (52.5)	48/85 (56.5)	0.54
Duration of HIV infection, years			0.778
Median (IQR)	17.0 (8.0, 25.0)	16.0 (7.0, 26.0)	
Range	(0.0, 38.0)	(0.0, 39.0)	
Missing/unknown	19 (9.6%)	3 (3.2%)	
Duration of HIV infection, years, n (%)			0.963
Unknown	19	3	
<10	50 (28.1)	26 (29.2)	
10–19	57 (32.0)	29 (32.6)	
20+	71 (39.9)	34 (38.2)	
CD4 nadir (cells/mm^3^)			0.161
Median (IQR)	220.0 (120.0, 400.0)	293.0 (146.5, 427.0)	
Range	(1.0, 900.0)	(10.0, 900.0)	
Unknown	72 (36.5%)	32 (34.8%)	
CD4 nadir (cells/mm^3^), n (%)			0.334
<100	27 (21.6)	7 (11.7)	
100–199	25 (20.0)	12 (20.0)	
200–299	27 (21.6)	12 (20.0)	
300–399	14 (11.2)	12 (20.0)	
≥400	32 (25.6)	17 (28.3)	
Unknown	72	32	
CD4 count (cells/mm^3^)			0.409
Median	620.0 (422.0, 855.0)	667.0 (446.0, 865.0)	
Range	(9.0, 1800.0)	(84.0, 1180.0)	
Missing	11 (5.6%)	9 (9.8%)	
CD4 count (cells/mm^3^), n (%)			0.763
<250	12 (6.5)	6 (7.2)	
250–349	15(8.1)	3 (3.6)	
350–499	38(20.4)	18 (21.7)	
500–999	100 (53.8)	46 (55.4)	
≥1000	21(11.3)	10 (12.0)	
Unknown	11	9	
CD4/CD8 ratio			0.048
Median (IQR)	0.80 (0.51, 1.15)	0.90 (0.60, 1.33)	
Range	(0.00, 2.50)	(0.15, 2.40)	
Missing	21 (10.7%)	12 (13.0%)	
CD4/CD8 ratio ≥ 0.75, n (%)	93/176 (52.8)	51/80 (63.8)	0.103
Undetectable viral load for at least 6 months, n (%)	177/195 (90.8)	77/89 (86.5)	0.279
ART regimen, n (%)			0.052
NRTI-based regimen	2 (1.0)	1 (1.1)	
NNRTI-based regimen	21 (10.7)	3 (3.3)	
PI-based regimen	6 (3.0)	2 (2.2)	
INSTI-based regimen	144 (73.1)	68 (73.9)	
Other ^3^	20 (10.2)	18 (19.6)	
None	4 (2.0)	0 (0.0)	
Paid or unpaid work in an environment where you work in close proximity to other people, n (%)	59/193 (30.6)	32/90 (35.6)	0.403
Working in hospital or healthcare facility, n (%)	9/193 (4.7)	3/90 (3.3)	0.605
Number of individuals (including participant) living in household, n (%)			0.268
1	98 (51.9)	39 (43.3)	
2	68 (36.0)	36 (40.0)	
3	17 (9.0)	8 (8.9)	
4 or more	6 (3.2)	7 (7.8)	
Unknown	8	2	
Number of bedrooms in household per person, mean (SD)	1.3 (0.7)	1.1 (0.6)	0.124
Number of bathrooms in household per person, mean (SD)	1.0 (0.5)	1.0 (0.5)	0.788

^1^ Multiple could be selected. ^2^ (CD4 < 350, CD4/CD8 < 0.75, suppressed VL). ^3^ Regimens containing combinations of the above and/or other drug classes (i.e., cell entry inhibitor). Comparisons were based on Chi-square test or Fisher’s exact test as appropriate. Other continuous variables, such as age and CD4 count, were compared between groups using Wilcoxon rank sum test.

**Table 2:** Types of vaccines received and time interval between doses in PWH without vs. with BTI.

	Breakthrough Infection No	Breakthrough Infection Yes	*p*
Types of COVID-19 vaccines received, doses 1 and 2, n (%)			0.004
mRNA–mRNA	182 (89.7)	71 (76.3)	
ChAdOx1–mRNA	13 (6.4)	9 (9.7)	
ChAdOx1–ChAdOx1	7 (3.4)	12 (12.9)	
Janssen/Novavax	1 (0.5)	1 (1.1)	
Types of COVID-19 vaccines received, dose 3, n (%)			0.929
Unknown/received only 2 doses	34	13	
BNT162b2	67 (67/163, 41.1)	32 (32/79, 40.5)	
mRNA-1273	96 (96/163, 58.9)	47 (47/79, 59.5)	
Number of vaccine doses received at the time of BTI, n (%)			-
2	-	25 (26.9)	
3	-	59 (63.4)	
4	-	9 (9.7)	
Number of days since last vaccine dose (IQR)	-	127.5 (67, 176)	-
Number of days since second dose (for persons who develop BTI between second and third doses) (n = 25)		168 (135, 182)	
Number of days since third dose (for persons who develop BTI between third and fourth doses) (n = 59)		108 (55, 167)	
Follow-up time from dose 2 to study end or BTI, months (median, IQR)	14.0 (11.5, 17.3)	8.8 (6.1, 10.9)	<0.001
Follow-up time from third dose (for those who did not have BTI prior to third dose)	8.8 (6.1, 12.4)	3.9 (1.9, 5.7)	<0.001
Follow-up time from fourth dose (for those who did not have BTI prior to fourth dose)	1.6 (1.0, 3.3)	0.6 (0.3, 1.4)	0.017

Comparisons were based on Chi-square test or Fisher’s exact test as appropriate. Other continuous variables, were compared between groups using Wilcoxon rank sum test.

**Table 3:** Adjusted odds ratio of breakthrough infection per log10 BAU/mL decrease in IgG.

Time Point ^1^		aOR (95% CI) ^2^	P^adj 3^
3 months post dose 2 (±1 month)	IgG spike	1.21 (0.73, 1.99)	0.464
	IgG RBD	1.19 (0.74, 1.91)	0.381
6 months post dose 2 (±2 months)	IgG spike	1.74 (0.95, 3.16)	0.071
	IgG RBD	1.71 (0.92, 3.19)	0.092
1 month post dose 3 (±2 weeks)	IgG spike	2.83 (1.02, 7.87)	0.046
	IgG RBD	2.84 (1.13, 7.15)	0.027

^1^ Only data prior to breakthrough infection were included. Participants who developed BTI prior to any of these time points were excluded from these time points. ^2^ Adjusted odds ratio of breakthrough infection per log10 BAU/mL decrease in IgG. ^3^ Logistic regression adjusted for age, sex, multimorbidity, hypertension, chronic kidney disease, diabetes and obesity was used to examine the effect of IgG spike/RBD on the odds of BTI.

## Data Availability

The data presented in this study may be made available upon written request to the corresponding author. Researchers wishing to access the CITF core data elements can submit an application to the CITF Data Access Committee.

## Supplementary Materials

The following supporting information can be downloaded at https://www.mdpi.com/article/10.3390/vaccines12050447/s1, Table S1: Participant characteristics stratified based on COVID-19 BTI; Table S2: COVID-19 symptoms based on presence or absence of BTI for participants self-reporting BTI; Table S3: Characteristics of the three participants with BTI who were hospitalized due to COVID-19.

## Appendix A

**Table A1:** Study period COVID-19 symptom questionnaire. Have you experienced any of the following COVID-19 signs or symptoms?

Sign	Yes	No	If Yes, Provide Date/Time
Fever, chills			
Cough			
Shortness of breath			
Acute loss of smell or taste			
Fatigue			
Headache			
Muscle aches			
Nausea/vomiting/diarrhea			
General weakness			
Nasal congestion			
Sore throat			
