# Supplementary material for: Correlates of Breakthrough SARS-CoV-2 Infections in People with HIV: Results from the CIHR CTN 328 Study
Source: Vaccines (Basel). 2024 Apr 23;12(5):447. doi: 10.3390/vaccines12050447 (PMC11125718; doi:10.3390/vaccines12050447)
Supplement: Supplementary file 1 [file vaccines-12-00447-s001.zip › vaccines-2926934-supplementary.pdf]

Supplementary Tables

Table S1: Participant characteristics stratified based on COVID-19 BTI

	**Participants without COVID-19 BTI (n=197)**	**Participants with COVID-19 BTI (n=92)**	
**Variable**			P
**Medical Conditions**			
Cardiovascular disease	10 (5.2)	4 (4.3)	0.761
Chronic kidney disease	9 (4.7)	2 (2.2)	0.308
Hypertension	31 (16.1)	8 (8.7)	0.091
Diabetes	20 (10.4)	4 (4.3)	0.087
Dyslipidemia	30 (15.5)	11 (12.0)	0.420
Chronic lung disease	19 (9.8)	6 (6.5)	0.354
Chronic liver disease	9 (4.7)	6 (6.5)	0.511
Blood disorder	3 (1.6)	3 (3.3)	0.392
Endocrine disorder	9 (4.7)	6 (6.5)	0.511
Malignancy	7 (3.6)	2 (2.2)	0.512
Metabolic disturbance	5 (2.6)	4 (4.3)	0.428
Rheumatological condition	8 (4.1)	3 (3.3)	0.717
Vascular disease	2 (1.0)	0 (0.0)	1.000
Obesity	38/186 (20.4)	18/87 (20.7)	0.961
**Body mass index**			0.961
Median (IQR)	26.1 (23.3, 29.2)	26.2 (23.2, 29.1)	
Range	(16.1, 58.7)	(15.5, 44.8)	
Number missing	11	5	
**Tobacco smoker**	47 (24.4)	16 (17.4)	0.185
**Use e-cigarettes**	10 (5.2)	0 (0.0)	0.027
**Mode of HIV acquisition**			
Sex with a man	127 (64.5)	65 (70.7)	0.300
Sex with a woman	18 (9.1)	6 (6.5)	0.453
Injection drug use	12 (6.1)	8 (8.7)	0.416
Blood product	2 (1.0)	3 (3.3)	0.331
Occupational exposure	0 (0.0)	0 (0.0)	-
Perinatal transmission	3 (1.5)	1 (1.1)	1.000
Other	8 (4.1)	1 (1.1)	0.175
Unknown	41 (20.8)	14 (15.2)	0.259
**Risk factors for HIV acquisition**			
Injection drug use	17 (8.6)	8 (8.7)	0.985
Blood product recipient	4 (2.0)	3 (3.3)	0.683

MSM	105 (53.3)	51 (55.4)	0.734
Bisexual	8 (4.1)	4 (4.3)	0.909
Originating from an endemic country	25 (12.7)	10 (10.9)	0.659
Sex work	2 (1.0)	1 (1.1)	1.000
Hemophilia	0 (0.0)	1 (1.1)	0.318
Other	18 (9.1)	3 (3.3)	0.073
Unknown	39 (19.8)	23 (25.0)	0.315
**AIDS Defining Illness, n (%)**			
None	157 (79.7)	77 (83.7)	0.420
Candidiasis, esophageal	3 (1.5)	2 (2.2)	0.655
Cryptococcosis, extrapulmonary	0 (0.0)	1 (1.1)	0.318
Cytomegalovirus disease (other than liver, spleen, or nodes)	1 (0.5)	1 (1.1)	0.536
Cytomegalovirus retinitis (with loss of vision)	2 (1.0)	0 (0.0)	1.000
Encephalopathy, HIV-related	3 (1.5)	1 (1.1)	1.000
Herpes simplex: chronic ulcer(s) (greater than 1 month's duration); or bronchitis, pneumonitis or esophagitis	1 (0.5)	1 (1.1)	0.536
Histoplasmosis, disseminated or extrapulmonary	1 (0.5)	0 (0.0)	1.000
Kaposi's sarcoma	8 (4.1)	2 (2.2)	0.414
Lymphoma, Burkitt's (or equivalent term)	3 (1.5)	3 (3.3)	0.387
Mycobacterium avium complex M. kanasii, disseminated or extrapulmonary	1 (0.5)	1 (1.1)	0.536
Mycobacterium tuberculosis, any site (pulmonary or extrapulmonary)	2 (1.0)	1 (1.1)	1.000
Pneumocystis carnii pneumonia	14 (7.1)	2 (2.2)	0.088
Pneumonia, recurrent	5 (2.5)	0 (0.0)	0.182
Toxoplasmosis of brain	2 (1.0)	0 (0.0)	1.000
Wasting syndrome due to HIV	3 (1.5)	2 (2.2)	0.655
Other	7 (3.6)	4 (4.3)	0.742

**Hospitalized in the 12 months preceding study enrolment, n (%)**	16/194 (8.2)	6/92 (6.5)	0.609
**Cannabis usage in the past 12 months, n (%)**	60/193 (31.1)	42/92 (45.7)	0.016
**Highest level of education completed, n (%)**			0.508
Prefer not to answer/No response	10	2	
Less than high school graduation	15 (8.0)	5 (5.6)	
High school graduation	47 (25.1)	17 (18.9)	
Trade certificate, vocational school, or apprenticeship training	20 (10.7)	9 (10.0)	
Non-university certificate or diploma from a community college, CEGEP	40 (21.4)	17 (18.9)	
University Bachelor’s degree	40 (21.4)	28 (31.1)	
University graduate degree (Master’s, Doctorate, etc.)	25 (13.4)	14 (15.6)	

Table S2: Symptoms reported by participants with BTI. Note that data was only available for 69/92 participants with BTI.

Signs, n (%)	All BTI (n=69)
Fever, chills	32 (46.4)
Cough	37 (53.6)
Shortness of breath	12 (17.4)
Acute loss of smell or taste	12 (17.4)
Fatigue	24 (34.8)
Headache	29 (42.0)
Muscle aches	20 (29.0)
Nausea/Vomiting/Diarrhea	10 (14.7)
General weakness	19 (27.5)
Nasal congestion	22 (31.9)
Sore throat	29 (42.0)

Table S3. Characteristics of the 3 participants with BTI who were hospitalized due to COVID-19

ID	Age	Sex	Multimorbidity	CD4 count
102087	56	F	N	1108
208002	65	M	Y	343
401050	59	M	Y	1090
